# Intimate partner violence and psychosocial health, a cross-sectional study in a pregnant population

**DOI:** 10.1186/s12884-015-0710-1

**Published:** 2015-11-11

**Authors:** An-Sofie Van Parys, Ellen Deschepper, Kristien Michielsen, Anna Galle, Kristien Roelens, Marleen Temmerman, Hans Verstraelen

**Affiliations:** Department of Obstetrics and Gynaecology, Faculty of Medicine and Health Sciences, International Centre for Reproductive Health, Ghent University, De Pintelaan 185, UZP 114, 9000 Ghent, Belgium; Department of Public Health, Biostatistics Unit, Faculty of Medicine and Health Sciences, Ghent University, Ghent, Belgium; Department of Obstetrics and Gynaecology, Faculty of Medicine and Health Sciences, Ghent University, Ghent, Belgium

**Keywords:** Intimate partner violence, Abuse, Pregnancy, Psychosocial health

## Abstract

**Background:**

The objective of this paper is to explore whether IPV 12 months before and/or during pregnancy is associated with poor psychosocial health.

**Methods:**

From June 2010 to October 2012, a cross-sectional study was conducted in 11 antenatal care clinics in Belgium. Consenting pregnant women were asked to complete a questionnaire on socio-demographics, psychosocial health and violence in a separate room. Overall, 2586 women were invited to participate and we were able to use data from 1894 women (73.2 %) for analysis. Ethical clearance was obtained in all participating hospitals.

**Results:**

We found a significant correlation between IPV and poor psychosocial health: within the group of women who reported IPV, 53.2 % (*n* = 118) had poor psychosocial health, as compared to 21 % (*n* = 286) in the group of women who did not report IPV (*P* < 0.001).

Lower psychosocial health scores were associated with increased odds of reporting IPV (aOR 1.55; 95 % CI 1.39–1.72), with adjustments made for the language in which the questionnaire was filled out, civil/marital status, education and age. In other words, a decrease of 10 points on the psychosocial health scale (total of 140) increased the odds of reporting IPV by 55 %.

When accounting for the 6 psychosocial health subscales, the analysis revealed that all subscales (depression, anxiety, self-esteem, mastery, worry and stress) are strongly correlated to reporting IPV. However, when accounting for all subscales simultaneously in a logistic regression model, only depression (aOR 0.87; 95 % CI 0.84–0.91) and stress (aOR 0.85; 95 % CI 0.77–095) remained significantly associated with IPV. The association between overall psychosocial health and IPV remained significant after adjusting for socio-demographic status.

**Conclusion:**

Our research corroborated that IPV and psychosocial health are strongly associated. Due to the limitations of our study design, we believe that future research is needed to deepen understanding of the multitude of factors involved in the complex interactions between IPV and psychosocial health.

**Electronic supplementary material:**

The online version of this article (doi:10.1186/s12884-015-0710-1) contains supplementary material, which is available to authorized users.

## Background

Intimate partner violence (IPV) is currently recognised as a global health problem with serious clinical and societal implications, which affects women and men from all backgrounds, regardless of age, ethnicity, socio-economic status, sexual orientation or religion [1–4]. IPV is defined as any behaviour within a present or former intimate relationship that leads to physical, sexual or psychological harm, including acts of physical aggression, sexual coercion, psychological abuse and controlling behaviour patterns [5]. IPV is also known as domestic/family violence, spouse/partner abuse/assault, battering, violence against women or gender-based violence [6–8]. Based on the Centers for Disease Control and Prevention’s definition of IPV [9], we have chosen to consistently use the term ‘violence’ for physical and sexual types of violence, and ‘abuse’ for psychological types. The word ‘abuse’ clearly refers to a broader range of behaviours than the word ‘violence’, which is often associated with severe forms of violent behaviour.

Pregnancy and childbirth mark an important turning point at which the roles and relationships of couples and their families are redefined on different levels. While parenthood can bring joy, it also confronts couple relationships with new challenges [10, 11]. As pregnancy may generate changes in physical, emotional, social and economic needs, it can be a stressful time. This period is associated with increased demands on individual capacities, the intimate partner relationship and household economic resources, and a reduction in leisure time and opportunities to socialise, which can exert adverse effects on emotional wellbeing [10]. Individual and dyadic coping strategies tend to decrease under stress, leading to an increased risk of physical and psychological aggression [12–14]. The vulnerable period for IPV associated with pregnancy extends further than the time between conception and birth - from a year before conception until one year after childbirth [4, 12–15].

A wide range of prevalence rates, from 3 to 30 %, have been reported for IPV around the time of pregnancy. Prevalence rates are mainly situated at the high end of the continuum in African and Latin American countries, and at the lower end in European and Asian countries. Although estimates are highly variable due to methodological challenges, the majority of studies show rates within the range of 3.9 to 8.7 % [3, 4, 6, 8, 10–17]. Although the exact prevalence of IPV around the time of pregnancy remains unclear, it is evident that it affects a substantial group of women. In Belgium, we recently showed [17] that as many as 15.8 % (95 % CI 14.2–17.7) of women experience IPV (incl. psychological abuse) before and/or during pregnancy. In other words IPV during the perinatal period is more common than several maternal physical health conditions (e.g. pre-eclampsia, placenta praevia), yet IPV receives considerably less attention within perinatal care [3, 4, 18, 19]. The Belgian perinatal health care system is based on the bio-medical model [20] with obstetrician/gynaecologists (ob/gyn’s) not only accounting for obstetric and gynaecologic pathology, but also acting as primary care physicians to the general female population, e.g. in providing primary obstetric care and in offering preventive women's health medicine [16, 21]. Although pregnancy brings women into regular contact with the health care system and therefore offers strategic opportunities to identify and ameliorate psychosocial concerns and risk factors [22], screening or systematic inquiry for IPV and/or psychosocial health is not part of routine perinatal care (yet).

In recent decades, research from the Western world, and increasingly, from low and middle income countries [23], has generated growing evidence that violence is associated with detrimental effects on the physical health of women, men and children, such as infection, miscarriage/abortion, placental abruption, foetal injury and perinatal death [8, 18, 19, 24–35]. Evidence is emerging that on the one hand, poor psychosocial health is a negative consequence of IPV, and on the other hand, poor psychosocial health is simultaneously found to be a risk factor for IPV. Moreover, poor psychosocial health status is linked to adverse pregnancy outcomes. Women reporting depressive symptoms and poor overall psychosocial health during pregnancy are at increased risk of low birth weight (LBW) and preterm birth [36]. Furthermore, reporting IPV, is associated with increased risk for anxiety disorders, eating disorders, anxiety attacks, nervousness, concentration problems, sexual dysfunctions, fear of intimacy, loss of self-esteem, psychosomatic complaints (e.g. headaches), pre- and postnatal depression, trauma symptoms (such as sleeping problems, flashbacks, panic attacks) post-traumatic stress syndrome, postpartum psychosis, and (attempted) suicide [18, 19, 24–35]. Additionally, IPV is strongly linked with harmful health behaviours such as using tobacco, alcohol or illicit drugs, poor maternal nutrition, and high-risk sexual behaviour [2, 8, 18, 19, 24–28, 31–35, 37–41].

The objective of this paper is to explore whether IPV 12 months before and/or during pregnancy is associated with poor psychosocial health in Flanders, Belgium.

## Methods

### Setting/study population

We conducted a multi-centre cross-sectional study in Flanders, the Northern part of Belgium. The Belgian perinatal health care system is based on the medical model [20] and is generally considered to be highly accessible, with women choosing their own health care provider(s). Obstetricians/gynaecologists (OB/GYN) merely function as primary perinatal health care providers and the majority of the care is hospital-based. Screening or systematic inquiry for IPV is not part of routine perinatal care.

This study was part of an RCT (Randomized Controlled Trial) that aimed to assess the impact of an intervention on psychosocial health, IPV, safety- and help-seeking behaviour. The methods have been previously published [20] and will only be summarized here.

Participants were recruited between June 2010 and October 2012 in 11 antenatal care clinics that were selected through a convenience sample (based on geographic location, including rural and urban settings, small and large hospitals). The selection criteria for participants were: being pregnant, minimum 18 years old and able to fill out a Dutch, French or English questionnaire. Overall, 2586 women were invited to participate and we were able to use data from 1894 women (73.2 %) for analysis. The study was introduced by the midwife or receptionist as a survey on difficult moments and feelings during pregnancy. Informed consent was obtained from all participants and consenting women were invited to fill out the questionnaire in a separate room without any accompanying person present. The questionnaire was returned to the health professional in a coded and sealed envelope. If the woman was unable to fill in the questionnaire in private, she was excluded from the study for safety reasons. All measures were taken to ensure that women could get additional support (from social services) if this was deemed necessary by the respondent or the staff. The information letter clearly indicated that the aim of the study was not to provide support or guidance. If women needed additional support (after filling out the questionnaire), they were referred to a 24/24 h telephone hotline. The involvement and training provided to the recruiting professionals was kept to a strict minimum since the aim of the RCT, of which this study was part, is to measure the effect of intervention in as unbiased a way as possible. The study was approved by the Ethics Committee of Ghent University and local ethical clearance was obtained from all 11 participating hospitals (Ethisch Comité Middelheim Ziekenhuis Netwerk Antwerpen, Ethisch Comité Universitair Ziekenhuis Antwerpen, Ethisch Comité Onze Lieve Vrouw Ziekenhuis Aalst, Ethisch Comité Gasthuis Zusters Ziekenhuis St Augustinus Antwerpen, Ethisch Comité Algemeen Ziekenhuis Sint Jan Brugge, Ethisch Comité Algemeen Ziekenhuis Jan Palfijn Gent, Ethisch Comité Onze Lieve Vrouw van Lourdes Ziekenhuis Waregem, Ethisch Comité Universitair Ziekenhuis Gent, Ethisch Comité Algemeen Ziekenhuis Groeninge Kortrijk, Ethisch Comité Virga Jesse Ziekenhuis Hasselt, Ethisch Comité Ziekenhuis Oost-Limburg Genk) (Belgian registration number 67020108164). The trial was registered at www.clinicaltrials.gov, identifier (NCT01158690).

The overall response rate was 76.7 %.

**Fig. 1:**
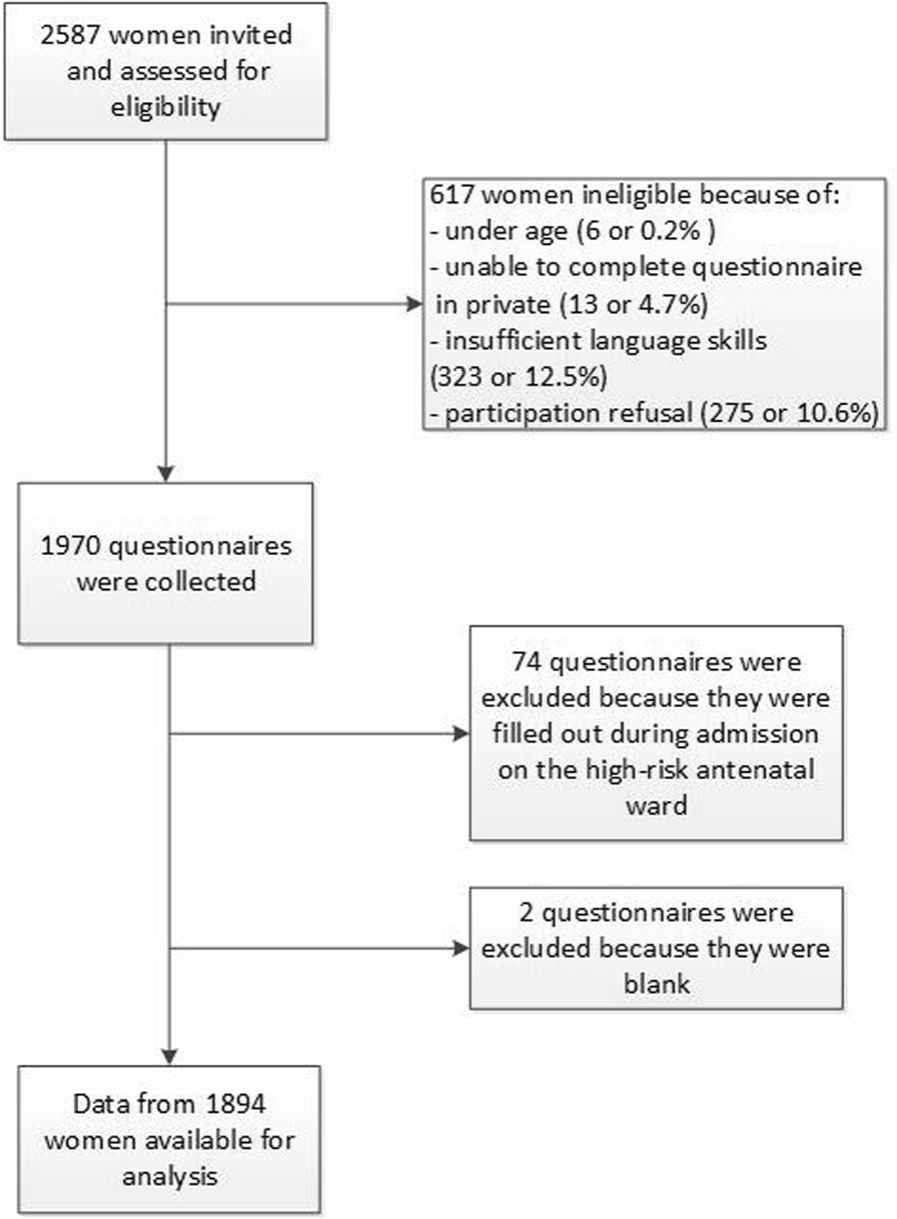
Flow diagram recruitment

Figure 1 provides a flow diagram of the recruitment.

### Questionnaire/measures

The questionnaire consisted of four main parts: socio-demographics, psychosocial health, violence and satisfaction with care. This paper focuses on the correlation of IPV with psychosocial health, while results on IPV prevalence and the evolution of IPV 12 months before and during pregnancy were published in another paper [17].

Physical and sexual (partner) violence was measured through an adapted version of the Abuse Assessment Screen (AAS) [42], which was adapted in consultation with one of the authors (Prof. dr. Judith McFarlane). To measure psychological abuse, we used an adapted version of the WHO-questionnaire [6]. Based on the limited available literature [1, 6, 43–50] and after long debate and extensive consultations with several experts in the field, we constructed a 7-item scale of questions with answer options ranging from 0 to 4 and we decided to use a cut-off value of 4/28 as a threshold for psychological abuse. We previously documented the assessment of abuse in detail [17]. Our scale had good internal consistency, with a Cronbach’s α value of 0.85 for 12 months before pregnancy and of 0.83 during pregnancy. For the purpose of this paper, we used a dichotomised variable including physical and/or sexual and/or psychological partner violence 12 months before pregnancy and/or during pregnancy.

Psychosocial health was measured through the Abbreviated Psychosocial Scale [51]. This scale is composed of 5 existing scales, namely, for trait anxiety (Speilberger Trait Anxiety Scale), self-esteem (Rosenberg Self-Esteem Scale), mastery (Pearlin Mastery Scale), depression (Centre for Epidemiologic Studies Depression Scale) and subjective stress (Schar Subjective Stress Scale). The Abbreviated Psychosocial Scale is well-validated and was recently identified as the best currently available instrument for measuring multiple psychopathological symptoms [52]. It consists of 6 subscales: negative affect (depression), positive affect (anxiety), positive self-esteem, low mastery, worry (anxiety) and stress. The scale consists of 28 questions, with response alternatives scored from 1 to 5, resulting in a minimum score of 28, indicating poor psychosocial health, and a maximum score of 140, indicating good psychosocial health. If one answer is missing, the overall score is coded as missing a value. Unfortunately, no clear clinical cut-off values for psychosocial health are currently available. Therefore, most authors [36, 51–55] use the median or P25-value as a threshold to dichotomize the scale into ‘poor’ or ‘good’ psychosocial health. Due to the lack of a clinical cut-off value, we used the scale as a continuous variable where possible. The scale has a Cronbach’s α of 0.93, indicating a high degree of reliability and internal consistency.

#### Data analysis

A descriptive analysis of socio-demographic variables, IPV and psychosocial health was performed. The bivariate correlation between IPV and psychosocial health was explored using the Pearson chi^2^ test. Binary logistic regression analysis was used to investigate the unadjusted and adjusted odds ratios (95 % confidence intervals) of reporting IPV correlated to psychosocial health (total score and subscale scores). Model selection was based on best model fit, statistical significance levels and clinical relevance. P- values below 0.05 were considered to be statistically significant. All statistical analyses were performed with IBM SPSS statistics software (version 22).

This research adhered to the STROBE guidelines for cross-sectional studies as outlined in http://www.strobe-statement.org/fileadmin/Strobe/uploads/checklists/STROBE_checklist_v4_cross-sectional.pdf (checklist added as Additional file 1).

## Results

### Socio-demographic data

The mean age of the women in our sample (*n* = 1894) was 28.9 years (SD 4.5) and the median gestational age was 23.9 weeks (IQR: 19–30). The large majority (95 %) of the women were married or living together with their partners; 5 % were divorced, separated or single. Sixty-two percent had completed higher education and 37.8 % had not. Most women (97.5 %) chose to fill out the questionnaire in Dutch, 0.9 % in French and 1.6 % in English. More details are presented in Table 1.

**Table 1:** Socio-demographic characteristics of sample (*n* = 1894)

Characteristics	Frequency (n)	Percent
Age (*n* = 1842) - years		
15–19	31	1.7
20–24	262	14.2
25–29	742	40.3
30–34	626	34.0
35–39	149	8.1
40–44	31	1.7
45–49	1	0.1
Civil/marital status (*n* = 1880)		
Married	928	49.4
Living together	857	45.6
Divorced or separated	13	0.7
Single	82	4.4
Education (*n* = 1878)		
None	34	1.8
Primary education	76	4.0
Secondary education	601	32.0
Non-university higher education	800	42.6
University higher education	367	19.5
Language questionnaire (*n* = 1894)		
Dutch	1846	97.5
French	17	0.9
English	31	1.6

### IPV prevalence

The overall percentage of IPV 12 months before and/or during pregnancy was 15.8 % (95 % CI 14.2–17.7) (*n* = 270), while it was 14.3 % (95 % CI 12.7–16.0) (*n* = 246) 12 months before pregnancy, and 10.6 % (95 % CI 9.2–12.1) during pregnancy, as we have previously reported in detail [17]. Physical partner violence before as well as during pregnancy was reported by 2.5 % (95 % CI 1.8–3.3) of the respondents, sexual violence by 0.9 % (95 % CI 0.5–1.4), and psychological abuse by 14.9 % (95 % CI 13.3–16.7). The proportion of missing values ranged between 4 % (*n* = 75) for physical and sexual violence and 10.2 % (*n* = 193) for psychological abuse.

### Psychosocial health

The median score for psychosocial health in our sample was 111 (IQR: 100–120), with a range from 55 to 140. The proportion of missing values was 10.1 %.

As noted above, the psychosocial health scale consists of 6 subscales: negative affect (depression), positive affect (anxiety), positive self-esteem, low mastery, worry (anxiety) and stress. Table 2 provides an overview of the subscale scores for the total population.

**Table 2:** Overview subscales psychosocial health

	Subscale depression	Subscale anxiety	Subscale self-esteem	Subscale mastery	Subscale worry	Subscale stress
Median total sample (IQR)	28 (25–31)	24 (23–27)	16 (15–18)	24 (21–27)	11 (9–13)	7 (5–8)
Median score women reporting IPV (IQR)	25 (21–28)	23 (20–24)	16 (14–17)	22 (18–24)	10 (8–11)	6 (5–7)
Median scores women not reporting IPV (IQR)	29 (25–31)	25 (23–27)	16 (15–18)	25 (22–27)	11 (10–13)	7 (6–8)
Maximum score subscales	35	30	20	30	15	10

### Correlation of IPV and psychosocial health

The bivariate analysis demonstrated a statistically significant correlation between IPV and psychosocial health. Within the group of women that reported IPV, 53.2 % (*n* = 118) had poor psychosocial health scores, as compared to 21 % (*n* = 286) in the group of women that did not report IPV (*P* < 0.001). Conversely, it can be stated that 29.2 % (*n* = 118) of the women with poor psychosocial health reported IPV, whereas 8.8 % (*n* = 104) of women with good psychosocial health reported IPV (*P* < 0.001).

### Correlation between psychosocial health, socio-demographics and IPV

Using a multivariable model, we found that a lower total psychosocial health score was associated with increased odds of reporting IPV (aOR 1.04; 95%CI 1.03–1.06), adjusted for the language in which the questionnaire was filled out, civil/marital status, education and age. This correlation means that a decrease of only one point on the total psychosocial health scale of 140 points is associated with an increased adjusted odds of reporting IPV of 4 %. In other words, a decrease of 10 points on the scale is associated with an increased adjusted odds of reporting IPV of 55 % (aOR 1.55; 95 % CI 1.39–1.72).

When accounting for the 6 psychosocial health subscales, as shown in Table 3, the binary analysis revealed that all psychosocial health subscales (depression, anxiety, self-esteem, mastery, worry and stress) were strongly correlated to reporting IPV. However, when accounting for all subscales simultaneously in a logistic regression model, only depression and stress remained significantly associated with IPV. The association between total psychosocial health and IPV remained significant after adjusting for socio-demographic status. All socio-demographic factors except age were significantly associated with reporting IPV.

**Table 3:** Association of psychosocial health with reporting IPV

Subscales psychosocial health	Unadjusted OR (95 % CI)	*P*-value	Adjusted OR (95 % CI)	*P*-value
Depression	0.83 (0.80–0.86)	<0.001	0.87 (0.84–0.91)	<0.001
Anxiety	0.82 (0.79–0.85)	<0.001	/	/
Self-esteem	0.86 (0.81–0.90)	<0.001	/	/
Mastery	0.84 (0.81–0.87)	<0.001	/	/
Worry	0.78 (0.73–0.83)	<0.001	/	/
Stress	0.73 (0.67–0.79)	<0.001	0.85 (0.77–095)	0.005
Total psychosocial health score	0.95 (0.94–0.96)	<0.001	/	/
Socio-demographics				
Age	/	/	1.00 (0.97–1.04)	0.700
Civil/marital status (single vs. married/cohabiting)	/	/	3.53 (1.89–6.60)	<0.001
Education				
No/primary vs. higher	/	/	5.62 (3.01–10.51)	<0.001
Secondary vs. higher	/	/	2.48 (1.78–3.45)	<0.001
Language questionnaire (not Dutch vs. Dutch)	/	/	5.75 (2.4–13.7)	<0.001

## Discussion

In this multi-centre cohort of pregnant women, we found a strong correlation between IPV and psychosocial health. Several other researchers have previously demonstrated a correlation between reporting IPV and poor psychosocial health [2, 8, 18, 24–28, 31–34, 38, 56–58]. Notably, poor psychosocial health is frequently reported as a negative consequence of IPV, and simultaneously, psychosocial health is found to be a risk factor for IPV. As this association has been repeatedly documented mostly in cross-sectional studies, it remains to be determined whether poor psychosocial health puts women at risk of IPV, or whether IPV induces worse psychosocial health, though it is plausible that both pathways co-exist. Literature on this specific matter is scarce; most studies have focussed on the association between poor psychosocial health and pregnancy outcomes such as low birth weight and prematurity, though the influence of psychosocial factors (such as stress, anxiety, and depression) on birth outcomes remains inconclusive [36, 51, 52]. However, psychosocial resources including self-esteem and mastery have been reported to protect women against stress from life events and chronic strains. These psychosocial resources could be even more relevant when women adapt to manage their lives and cope with the stress and vulnerability associated with IPV during pregnancy [54].

Our data further suggest that, after taking all measured variables into account, the correlation between IPV and psychosocial health was mainly explained by “depression” and “stress” as psychosocial health indices. It has been noted that scales measuring affective states such depression or anxiety are likely to be highly correlated with each other and measure generalized distress rather than symptoms unique to depression or anxiety [51]. Our results confirm the finding that there is a strong correlation between the different psychosocial health subscales. The strong association between the total psychosocial health scale and IPV might indeed refer to a more general form of distress in our population interconnected with a multitude of factors. Recently, there has been a shift towards envisaging psychosocial health as a multidimensional concept [52]. We acknowledge that psychosocial health is a complex construct with many known and, presumably, many unknown determinants, although our study was not designed to explore this. Future research should be done to try to shed some light on the multitude of factors involved in the complex interaction between psychosocial health and IPV.

Our results need to be viewed within the context of certain limits. There is currently a lack of agreement on standard measures for psychological (partner) abuse/violence and in an effort to tackle this problem, we decided to construct our own scale and threshold for psychological abuse cut-off value. The threshold we chose for psychological abuse was based on a thorough literature search and extensive discussions with experts in the field. Nevertheless, it remains an arbitrary choice that is open for discussion. We have some indication that the cut-off might be on the low side, but this hypothesis obviously needs further investigation. Furthermore, our study design did not allow us to determine causal pathways between the factors analysed. Moreover, we were not able to analyse in depth the multitude of factors involved in the complex interaction between IPV and psychosocial health, and as a consequence, might have oversimplified reality. The findings presented in this paper are based on a sample of the Belgian obstetrical population and cannot be generalised to other populations or health care systems without the necessary caution.

## Conclusion

Our research has demonstrated that IPV and psychosocial health are strongly associated. Due to the cross-sectional nature of our study design, we are not able to make any statements on causality with regard to these associations. However, it seems reasonable that a multitude of factors could have influenced the interaction, and more longitudinal and in-depth, qualitative analysis needs to be done to shed light on the complex interactions and confounding factors that define the relationship between IPV and psychosocial health.

Furthermore, linked to the important role of psychosocial health found in our study, we believe that the recommendation to routinely screen for IPV during pregnancy should be broadened and that IPV should not been seen as an isolated theme. IPV research is providing increasing evidence that addressing the multitude of risk factors related to IPV simultaneously has a larger effect than addressing a single factor. Therefore, we would like to join the growing number of authors advocating for the inclusion of IPV within a broader psychosocial health assessment as a standard part of antenatal care. Addressing psychosocial health in antenatal care has the potential to improve the health and well-being of women and their families.

## Additional file

Additional file 1:
**STROBE Statement—Checklist of items that should be included in reports of** ***cross-sectional studies.*** (PDF 18 kb)

## Abbreviations

AAS: Abuse Assessment Screen
IPV: Intimate Partner Violence
IQR: Inter Quartile Range
OB/GYN: Obstetrician/Gynaecologist
RCT: Randomized Controlled Trial
SD: Standard Deviation
SES: Socio Economic Status
SPSS: Statistical Package for the Social Sciences.
